# Beyond Clozapine Failure: Precision Approach and Palliative Psychiatry in Ultra-Treatment-Resistant Schizophrenia

**DOI:** 10.3390/jcm15187264

**Published:** 2026-09-18

**Authors:** Marc Peraire, Anna Moreno-Beltrán, Aitana Fillol, Mar Cabañés-Suñer, Rita Gimeno-Vergara

**Affiliations:** 1 Department of Mental Health, Consorci Hospitalari Provincial Universitari de Castelló, 12002 Castelló de la Plana, Spain; annmor96@gmail.com (A.M.-B.); aitanafillol@gmail.com (A.F.); marsuner@gmail.com (M.C.-S.); ritagimeno27@gmail.com (R.G.-V.); 2 TXP Research Group, Universidad Cardenal Herrera-CEU, 12006 Castelló de la Plana, Spain; 3 Fundación Hospital Provincial, 12002 Castelló de la Plana, Spain; 4 Pre-Departmental Medicine Unit, Universitat Jaume I, 12006 Castelló de la Plana, Spain

**Keywords:** treatment-resistant schizophrenia, clozapine-resistant schizophrenia, ultra-treatment-resistant schizophrenia, clozapine, electroconvulsive therapy, precision psychiatry, palliative psychiatry

## Abstract

Clozapine is the only evidence-based treatment for treatment-resistant schizophrenia (TRS); however, up to 70% of patients have an inadequate response, leading to clozapine-resistant schizophrenia (CRS) and, in its most severe form, ultra-treatment-resistant schizophrenia (UTRS). Because evidence-based treatment algorithms after clozapine failure remain limited, management requires an individualized, multimodal approach. We present a UTRS complex case alongside a scoping review conducted according to the PRISMA-ScR framework to summarize the current evidence on the diagnosis, neurobiology, and treatment of CRS and UTRS. A 24-year-old man with schizophrenia required 11 months of psychiatric hospitalization due to persistent psychosis, severe aggression, disorganized thinking, prominent negative symptoms, and profound functional impairment. Extensive investigations, including neuroimaging, cerebrospinal fluid analysis, metabolic studies, and genetic testing, ruled out alternative diagnoses. Despite sequential treatment with multiple antipsychotics, optimization of long-acting injectable therapy, clozapine, pharmacological augmentation, two courses of electroconvulsive therapy (ECT), and intensive multidisciplinary rehabilitation, the patient continued to exhibit severe positive, negative, and cognitive symptoms and functional impairment, meeting the criteria for UTRS. This review emphasizes the importance of excluding pseudoresistance by confirming adherence, assessing clozapine exposure when therapeutic drug monitoring is available, and conducting diagnostic reassessment before establishing a diagnosis of CRS or UTRS. Among available interventions, ECT remains the strongest evidence-based augmentation strategy following clozapine failure, while pharmacological augmentation and other neuromodulatory techniques are supported by low-certainty evidence. These findings underscore the need for individualized multimodal management that integrates pharmacological, biological, and psychosocial interventions, and support consideration of precision psychiatry and palliative psychiatry principles as potential future frameworks when evidence-based therapeutic options have been exhausted.

## 1. Introduction

Schizophrenia is a chronic and disabling psychiatric disorder associated with marked functional impairment, premature mortality, and a substantial social and economic burden [1,2]. Although multiple antipsychotics are available, approximately one-third of patients do not achieve adequate symptom control despite at least two trials with appropriate antipsychotics and are classified as having treatment-resistant schizophrenia (TRS) [2,3].

Clozapine is the only antipsychotic with established superiority in TRS and is recommended as the first-line treatment in major international guidelines [4], with additional evidence suggesting mood-stabilizing and anti-craving effects [5]. Nevertheless, its initiation is often delayed, and up to 70% of treated patients experience only partial or no clinical improvement despite adequate treatment, giving rise to the concepts of clozapine-resistant schizophrenia (CRS) and ultra-treatment-resistant schizophrenia (UTRS) [3,6,7].

CRS and UTRS are sometimes used interchangeably, but they represent different stages of treatment resistance. CRS refers to persistent psychotic symptoms despite an adequate clozapine trial, whereas UTRS describes patients who remain severely symptomatic despite clozapine optimization and subsequent evidence-based augmentation strategies [8]. This distinction requires consideration of the clozapine dose and treatment duration, adherence, therapeutic drug monitoring data, and the adequacy and duration of subsequent augmentation strategies. These patients typically have persistent positive symptoms, severe negative symptoms, cognitive impairment, profound functional disability, recurrent hospitalizations, and complex medication regimens that often provide limited benefit [2,6].

Before confirming clozapine resistance, potentially reversible causes of pseudoresistance—including poor adherence, inadequate plasma concentrations, insufficient treatment duration, substance use, and medical comorbidities—should be systematically excluded [2,9]. Once genuine resistance has been established, management requires a multimodal approach. Among biological interventions, electroconvulsive therapy (ECT) has the strongest evidence for augmentation, whereas pharmacological augmentation strategies have generally demonstrated inconsistent or modest benefits [10,11].

Despite increasing interest in refractory schizophrenia, substantial heterogeneity in diagnostic definitions, treatment protocols, and outcome measures limits comparability across studies and hinders the development of robust therapeutic recommendations. This scoping review summarizes the current evidence on pharmacological, biological, psychosocial, and palliative interventions for CRS and UTRS, using a complex clinical case to illustrate the challenges of individualized, multimodal care.

## 2. Materials and Methods

This clinical case was selected and described by the mental health professionals who provided the patient’s clinical care. For this purpose, consent was obtained from the patient to review data from his medical records.

Subsequently, a scoping review was conducted in accordance with the PRISMA-ScR Statement. The literature search was conducted on 31 July 2026, in Web of Science, PubMed, and Embase. This database combination was selected because it has been reported to retrieve approximately 96% of relevant articles [12].

The search strategy was based on two conceptual blocks: (1) clozapine-resistant schizophrenia or ultra-treatment-resistant schizophrenia; and (2) psychopathology, treatment, neurobiology, or biomarkers. The Boolean search structure was (clozapine-resistant schizophrenia OR ultra-treatment-resistant schizophrenia) AND (psychopathology OR treatment OR neurobiology OR biomarker). The terms were combined using the Boolean operators OR within each conceptual block and AND between the two blocks.

All terms were searched as free text. No date, publication-type, or other database filters were applied. In Embase, the exp field was used for the corresponding terms to expand the search to related indexed terms and narrower concepts. The electronic search strategy used is provided in the Supplementary Material (Table S1).

**Figure 1:**
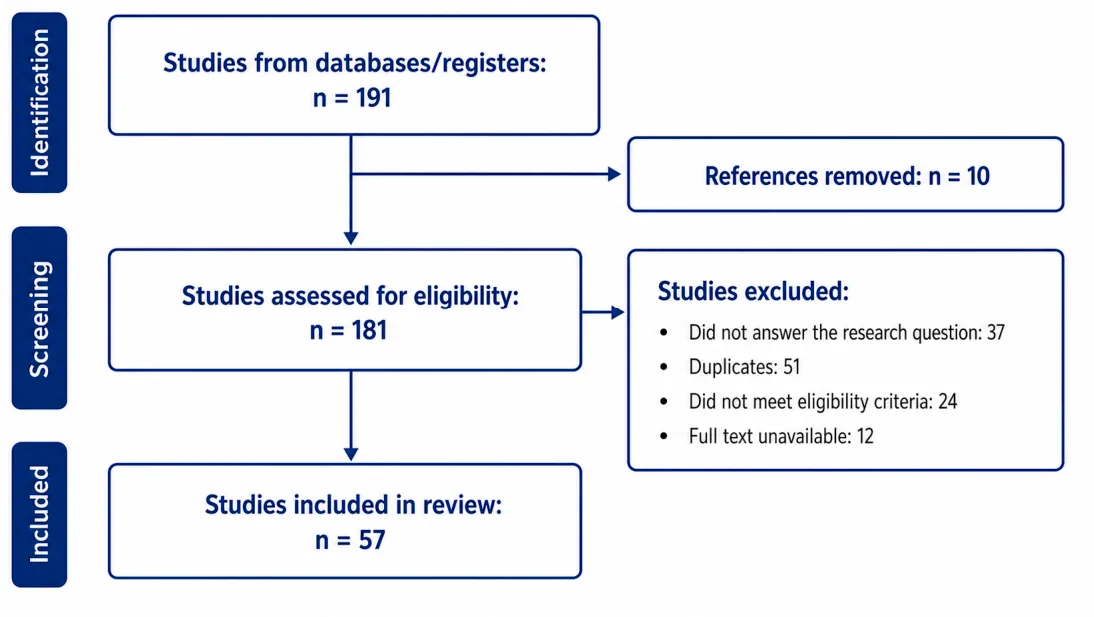
Flow diagram of study selection.

The article selection process is shown in Figure 1. Two reviewers (AF, MC) independently assessed the retrieved records according to the inclusion and exclusion criteria, and any resulting discrepancies were resolved by a third reviewer (MP).

Eligible publications included clinical trials, case reports, reviews, or meta-analyses that examined neurobiological features or treatment strategies in patients with CRS or UTRS. The exclusion criteria included studies that fell outside the scope of the review, such as those that only reported prevalence rates without examining the disease in depth, those focused exclusively on somatic or physical-health outcomes, or those that provided theoretical discussions of concepts without clinically relevant diagnostic or therapeutic content. Studies that were not in English or Spanish, that addressed a different study population, or for which the full text was unavailable were also excluded.

As this was an exploratory review designed to map the available evidence rather than estimate the comparative effects of treatments, no formal risk of bias or methodological quality assessment was conducted. However, the included publications were categorized according to their study design as meta-analyses and systematic reviews, randomized controlled trials, observational studies, case series, case reports, and narrative reviews or commentaries. This categorization was used to contextualize the findings and avoid interpreting preliminary evidence from uncontrolled or single-patient studies as equivalent to evidence from randomized trials or evidence syntheses.

## 3. Results

### 3.1. Case Presentation

The patient lived with both parents and a five-year-old older brother. He had normal psychomotor development, no relevant childhood or adolescent history, and good peer relationships. His premorbid personality was described as docile, polite, and affable, with no significant family psychiatric history.

At age 17, in April 2020, he developed persecutory delusions focused on his brother, whom he blamed for his academic difficulties. After briefly attending university, his delusional beliefs intensified following withdrawal from studies, extending to the conviction that household renovations were deliberately intended to disturb him and derail his future. Over the next two years, he became increasingly socially withdrawn, abandoned leisure activities, neglected self-care, and showed behavioral disturbance, suspiciousness, and suicidal ideation during the SARS-CoV-2 pandemic.

In September 2022, he was first admitted to the acute ward because of behavioral decompensation. He presented with delusional interpretations, self-referential speech, suspiciousness, and marked social withdrawal, with clear disruption of his autobiographical narrative. Antipsychotic treatment reduced delusional intensity and improved engagement and speech, although negative symptoms persisted. His PANSS and CGI scores were 121 and 7, respectively, at admission; these decreased to 85 and 4 at discharge. He was referred to Day Hospital, but poor insight and nonadherence limited progress, and he remained mainly on depot paliperidone.

During 2023, persecutory ideation persisted and gradually expanded to other family members and even the treating psychiatrist. Because oral adherence was poor, the depot dose was increased. Behavioral control subsequently improved, although psychotic symptoms persisted. Clozapine was introduced in May 2023, later combined with cariprazine, producing a temporary functional improvement with greater activity, regular routines, and some family contact. However, he soon disengaged from treatment again, discontinued medication, and remained isolated at home for more than a year, with predominantly negative symptoms and disorganized thinking.

After a prolonged period without treatment, he was readmitted with severe behavioral and psychotic worsening, including incoherent speech, religious and possession-related delusions, auditory hallucinations, and violent ideation. His course subsequently deteriorated further, with increasingly disorganized and incoherent speech, frequent agitation, echolalia, and repeated episodes requiring physical restraint. In late 2025, he required internal medicine admission for nutritional support due to a catatonic-like state with severe psychomotor slowing and tremor, followed by partial improvement after antipsychotic withdrawal and later reintroduction of partial agonists and low-dose antagonists. At the time of his second admission, his PANSS score was 192, and his CGI score was 7. Throughout the subsequent monthly assessments, the PANSS score consistently remained above 120, while the CGI score remained above 5.

By March 2026, he remained in long-term care with severe functional impairment, poverty of speech, cognitive decline, social isolation, religious and self-deprecating delusions, and recurrent psychomotor agitation. He was readmitted in April 2026 with further disorganization, sexual disinhibition, tremor, and bizarre speech, and was eventually transferred to a more restrictive long-stay setting, where he gradually adapted to the ward routine and no longer verbalized delusional content. During that stay, he was admitted with a PANSS of 175 and a CGI of 7, which were reduced at the time of discharge to 139 and 4. The case chronological evolution is summarized in Table 1.

In this case, clozapine was first initiated in 2023 but was subsequently discontinued by the patient during outpatient follow-up, after only 4 months. Following the patient’s second admission, clozapine was reintroduced in May 2025 and subsequently titrated to 300 mg/day, a dose maintained for 6 months. Although clozapine plasma concentrations were not systematically measured because therapeutic drug monitoring was not routinely available in our laboratory, adherence could be reliably confirmed: the patient remained hospitalized for almost a year without a therapeutic discharge, and medication administration was directly supervised throughout treatment. Thus, despite a history of poor adherence during previous outpatient treatment attempts, adherence during the clozapine trial was adequate and sustained. Moreover, the patient did not smoke or use other substances and did not receive concomitant medications likely to interfere with clozapine metabolism.

Despite adequate and sustained clozapine treatment, severe positive, negative, cognitive, and functional symptoms persisted. Clozapine was subsequently augmented with amisulpride at 1200 mg/day for 3 months, without sustained clinical benefit, and ECT was administered in two cycles of 10 sessions each, again without sustained improvement. During previous and subsequent periods, clozapine was also augmented with partial dopamine agonists, including cariprazine and aripiprazole, at therapeutic doses. Therefore, given the persistence of severe symptoms despite confirmed adherence, adequate clozapine exposure, optimization strategies, and subsequent pharmacological and ECT augmentation, the patient met criteria for genuine treatment resistance and was classified as UTRS.

### 3.2. Scoping Review

Table 2 summarizes the 57 studies included in the scoping review. Of these, 33 articles (57.9%) included CRS participants, 15 articles (26.3%) investigated UTRS, and the remaining 9 (15.8%) included samples of both disorders. The countries most frequently represented were India (9, 15.8%), the United States (6, 10.5%), Brazil (5, 8.7%), China (5, 8.7%), Italy (4, 7%), and Japan (4, 7%). The most frequent domains were pharmacological augmentation (19, 33.3%), neuromodulation (10, 17.5%), neurobiology (9, 15.8%), and ECT (7, 12.3%).

The included evidence was heterogeneous in both study design and clinical scope. Meta-analyses and systematic reviews accounted for 15 studies (26.3%); randomized controlled trials, 8 (14.1%); observational studies, 16 (28.0%); case series, 3 (5.2%); case reports, 8 (14.1%); and narrative reviews, 7 (12.3%) (Table S2). This distribution indicates that, despite the relatively large number of studies, only a minority of the included literature consisted of randomized controlled trials, while a substantial proportion comprised observational studies and uncontrolled reports. Consequently, these designs should not be treated as equivalent in evidential strength. Randomized controlled trials provide the strongest direct evidence of treatment efficacy, while observational studies provide supportive but potentially confounding evidence, and case series or case reports primarily provide hypothesis-generating signals. This distinction was particularly relevant for emerging pharmacological and neuromodulatory interventions, whose apparent clinical benefits often come from small, uncontrolled samples rather than comparative trials.

#### 3.2.1. Neurobiological Findings and Potential Biomarkers

Available neuroimaging studies suggest that CRS and UTRS may represent biologically distinct subtypes within treatment-resistant schizophrenia [50]. Reported structural and functional abnormalities have involved prefrontal, frontostriatal, cingulate and parietal networks, together with reduced cortical gyrification and altered functional connectivity [28,43,48,54].

Neurochemical studies have identified abnormalities in glutamatergic neurotransmission, including increased glutamate–glutamine concentrations in the dorsal anterior cingulate cortex and reduced putaminal glutamatergic metabolites in patients with UTRS [20,33]. Glutamatergic dysfunction, particularly reduced N-methyl-D-aspartate (NMDA) receptor signaling on GABAergic interneurons, may contribute to the pathophysiology of schizophrenia and treatment resistance by disrupting cortical network synchrony, cognition, negative symptoms, and downstream dopaminergic signaling [39]. This hypothesis is supported by the psychotomimetic and cognitive effects of NMDA receptor antagonists, as well as by neurochemical and magnetic resonance spectroscopy findings indicating altered glutamatergic metabolites in CRS and UTRS.

Preliminary studies have linked UTRS to peripheral markers, including glial fibrillary acidic protein (GFAP), neurofilament light chain (NfL), ubiquitin C-terminal hydrolase L1, inflammatory cytokines, and LINE-1 dysregulation [58,59]. Isolated reports have also described potentially relevant genetic variants, including a KMT2D variant (c.4168G>A; p.Ala1390Thr) [47].

#### 3.2.2. Pharmacological Augmentation Strategies

Within the evidence identified by our search, no pharmacological augmentation strategy has consistently demonstrated superiority after inadequate response to clozapine monotherapy [41]. The reviews and meta-analyses identified generally concluded that the overall quality of evidence remains low, with no intervention achieving Grade A recommendations [25,37].

Among antipsychotic augmentation strategies, the strongest evidence supports the addition of amisulpride, which has demonstrated improvements in overall psychopathology, positive symptoms, and general psychopathology, with generally acceptable tolerability [45,46,63]. Aripiprazole has also been evaluated in randomized and meta-analytic evidence and has shown potential benefits on psychopathology, metabolic parameters, and functional outcomes, both in oral [17,22] and long-acting injectable formulations [38]. Small studies and case reports have suggested potential benefits with cariprazine [49], brexpiprazole [44], and lurasidone [62], particularly regarding negative and cognitive symptoms, although the evidence is limited to small-sample studies and case reports.

Other pharmacological augmentation strategies targeting non-dopaminergic mechanisms have also been investigated. Glutamatergic and antioxidant approaches include sodium benzoate, which has been investigated as a potential augmentation strategy in CRS, although the available evidence remains limited [39,69]. More recently, muscarinic receptor modulation has emerged as a novel therapeutic approach. The xanomeline–trospium combination, a central M1/M4 muscarinic receptor agonist, xanomeline, and the restricted peripheral muscarinic antagonist, trospium, has demonstrated efficacy in randomized controlled trials of schizophrenia. However, specific evidence in CRS and UTRS remains extremely limited; in the present review, only two case reports evaluated the adjunctive xanomeline–trospium combination in CRS and reported limited clinical benefit [68].

Regarding non-antipsychotic adjunctive medication, lamotrigine has shown modest benefits in several meta-analyses [15,41,63], while memantine and mirtazapine have been consistently ranked among the most promising augmentation strategies for general and negative symptoms [30,42,53,55,63]. Other agents, including sodium valproate [30], fluoxetine and duloxetine [37], topiramate and ziprasidone [63], sodium benzoate [37,39,69], levothyroxine [21], and raloxifene [19], have shown positive results in selected studies, but remain supported by heterogeneous and generally low-certainty evidence.

Although polypharmacy may be clinically attractive because it is relatively easy to implement, its benefits are generally modest and it may increase the risk of adverse effects [18,35], both with clozapine combination therapy [13] and with the add-on second-generation antipsychotic [32].

#### 3.2.3. Electroconvulsive Therapy and Other Neuromodulation Techniques

ECT remains the intervention with the strongest available support among the augmentation strategies identified [53]. Several randomized trials, systematic reviews, and meta-analyses have shown greater improvement in psychotic symptoms, particularly persistent positive symptoms, when ECT is combined with clozapine compared to clozapine alone [26,27,36,42,51,65]. The consistency of these findings provides stronger support for ECT than for most pharmacological or other neuromodulatory approaches.

Some studies have reported clinically significant improvement in approximately half of patients receiving adjunctive ECT, and the response appears to be associated with the number of ECT sessions, stimulation intensity, and pre-treatment plasma clozapine concentrations [26]. Adverse effects are usually mild and reversible, consisting mainly of transient headache, confusion, and short-term memory impairment [35], although careful monitoring for seizures and cardiovascular complications remains necessary [23,67]. ECT may therefore be an important alternative when clozapine is ineffective or contraindicated [31,60].

Other neuromodulatory approaches remain under investigation. Repetitive transcranial magnetic stimulation has not consistently demonstrated superiority over sham [16,41]. Transcranial direct current stimulation (tDCS) has shown modest improvements in auditory hallucinations and global psychopathology [34], but its combination with clozapine is limited to case reports and requires confirmation in controlled trials [23]. Continuous theta burst stimulation (targeting lateral occipital cortex) [57,61], and transcranial alternating current stimulation (targeting dorsolateral prefrontal cortex) [24,56] have yielded encouraging preliminary results in small studies. Deep brain stimulation [66] and magnetic seizure therapy [64] also represent emerging therapeutic alternatives, with reported effects on positive symptoms and less cognitive impairment than ECT.

#### 3.2.4. Psychosocial Interventions and Overall Quality of Evidence

Beyond pharmacological and biological interventions, recovery-oriented psychosocial approaches, including cognitive remediation, family interventions, and intensive psychiatric rehabilitation, are consistently recommended for persistent negative symptoms and severe functional disability, although evidence specifically in CRS and UTRS remains scarce [40]. Cognitive behavioral therapy has yielded mixed results in randomized controlled trials, with uncertain clinical efficacy and limited cost-effectiveness [14,29].

Overall, the literature is highly heterogeneous, with inconsistent definitions of CRS and UTRS, variable criteria for clozapine exposure, heterogeneous outcome measures, and, generally, small-sample sizes. Although several pharmacological, biological, and psychosocial interventions show promise, the evidence remains insufficient to support a universally accepted treatment algorithm for clozapine-resistant or ultra-treatment-resistant schizophrenia [52].

## 4. Discussion

This case reflects the clinical complexity of patients who progress from TRS to CRS and, ultimately, to UTRS. Despite sequential treatment with multiple first-, second-, and third-generation antipsychotics, optimization of long-acting injectable medication, clozapine initiation, pharmacological augmentation with cariprazine, repeated courses of ECT, prolonged inpatient rehabilitation, and comprehensive diagnostic reassessment, the patient continued to exhibit severe positive, negative, cognitive, and functional symptoms. The clinical course illustrates the therapeutic challenges characteristic of UTRS, particularly the lack of a well-established treatment strategy after clozapine failure [2,40,70]. Importantly, given that multiple interventions were administered sequentially and concurrently, the observed clinical trajectory cannot be interpreted as evidence for or against the efficacy of any single treatment. Rather, the case illustrates the difficulty of drawing treatment-specific conclusions from complex treatment trajectories.

This complexity also underscores the importance of establishing genuine treatment resistance before diagnosing CRS or UTRS. Before considering clozapine ineffective, pseudoresistance must be excluded by confirming adherence, verifying therapeutic plasma concentrations, optimizing dose and duration, and identifying reversible factors such as substance use, medical comorbidity, or pharmacokinetic interactions [2,41,70]. Organic disorders that can mimic refractory psychosis should also be considered, especially when atypical neurological features or an unusual course are present [71]. In this case, extensive neuroimaging, cerebrospinal fluid analysis, metabolic studies, and genetic testing were performed before concluding that the patient met criteria for UTRS, confirming adherence and thereby reducing the likelihood of pseudoresistance. Distinguishing CRS from UTRS is also clinically relevant, as they represent different stages of treatment resistance [8,40]. Clozapine should be introduced early in TRS, whereas established CRS or UTRS generally requires individualized multimodal strategies rather than further antipsychotic switching; delayed clozapine initiation remains a barrier to optimal outcomes, and early use has been associated with better response [2,7,70,72,73].

Once clozapine has been optimized and genuine resistance confirmed, the pharmacological literature does not support a clearly superior augmentation strategy [42,63]. Although polypharmacy is common in routine practice [13,74], comparisons are limited by differences in definitions of resistance, clozapine exposure, symptom targets, outcome measures, and follow-up duration. Pharmacological augmentation should therefore be tailored to the clinical phenotype, previous treatment response, adverse-effect burden, comorbidities, and patient preferences rather than according to a universally accepted treatment hierarchy.

Among biological interventions, ECT remains the best-supported augmentation option after clozapine failure [51,67], particularly when persistent positive symptoms, catatonia, or severe behavioral disturbances predominate [27,53]. However, response is not consistent across patients, treatment protocols vary, and long-term functional outcomes remain insufficiently characterized [42,67]. Other neuromodulatory approaches remain investigational, with evidence derived mainly from small and heterogeneous studies [75]. Preliminary studies have reported possible benefits for auditory hallucinations, negative symptoms or overall psychopathology, but these findings require confirmation in larger controlled studies [23,34,41,61,66].

Our review also suggests that CRS and UTRS may represent biologically distinct forms of treatment resistance, rather than simply representing a more severe form of schizophrenia. Neuroimaging studies have identified abnormalities in prefrontal, frontostriatal, cingulate, and default mode networks, along with reduced cortical gyrification and altered functional connectivity [28,43,48,50,54]. Greater treatment resistance has also been associated with worse cognitive performance [76] and autistic traits [77]. In parallel, glutamatergic dysregulation, inflammatory markers, biomarkers of astroglial and neuroaxonal damage, epigenetic changes, and emerging genetic findings suggest pathophysiological mechanisms partially distinct from dopamine-responsive schizophrenia [20,33,58,59,78,79].

These findings provide a potential framework for precision psychiatry [80], which should currently be understood as a pragmatic framework for optimizing individualized treatment, rather than a validated approach to biomarker-guided treatment selection. Its most clinically applicable components currently include assessing adherence and adequate clozapine exposure through therapeutic drug monitoring, assessing pharmacokinetic interactions, and systematically characterizing symptomatic, cognitive, and functional profiles. Biomarkers, pharmacogenetics, and neuroimaging may assist diagnostic reassessment and exclusion of secondary causes and may contribute to future biological stratification. However, none has yet demonstrated sufficient validity, reproducibility, or clinical utility for routine treatment selection [81]. Such tools should complement, rather than replace, careful clinical assessment and shared decision-making.

Within this broader biological framework, the glutamatergic findings provide a plausible biological rationale for targeting NMDA receptor-related mechanisms in CRS and UTRS. Strategies involving glycine-site modulation, D-serine, and D-amino acid oxidase (DAO) inhibition have shown preliminary efficacy for negative and other symptom domains in schizophrenia when added to non-clozapine antipsychotics, but their efficacy has not been consistently demonstrated when combined with clozapine [82,83,84]. Sodium benzoate has also shown preliminary evidence of benefit in CRS [39,69]. Overall, enhancing NMDA receptor-mediated neurotransmission remains biologically plausible, but the gap between this rationale and demonstrated clinical efficacy in treatment-resistant populations remains substantial.

Xanomeline–trospium (KarXT) represents a promising non-dopaminergic approach targeting muscarinic M1/M4 receptors and expanding therapeutic strategies beyond conventional dopamine receptor modulation [85,86]. Randomized controlled trials have demonstrated antipsychotic efficacy in schizophrenia [87,88], but these findings have been obtained predominantly in broader schizophrenia populations rather than in patients with established clozapine resistance. In our review, evidence for xanomeline–trospium in CRS was limited to two case reports, both of which reported little clinical benefit [68]. Thus, although muscarinic strategies may offer a mechanistically distinct approach, their role in CRS and UTRS remains uncertain and requires further evaluation in well-characterized clozapine-resistant populations.

Importantly, management of UTRS extends beyond pharmacological and biological interventions. Multidisciplinary rehabilitation is a particularly important component of care in UTRS, as severe negative symptoms, cognitive impairment, and functional disability are unlikely to be adequately addressed by medication alone. Cognitive rehabilitation, family interventions, intensive psychosocial rehabilitation, and recovery-oriented care should therefore remain central to long-term management [89], although evidence specifically in UTRS is limited [6,40].

An important aspect of this case is the transition from a curative to a palliative therapeutic perspective. Psychiatry has traditionally focused on remission, but some patients continue to deteriorate despite comprehensive evidence-based care. In such situations, repeated escalation of treatment may increase side effects, coercive interventions, prolonged hospitalization, and loss of autonomy without a realistic prospect of further meaningful recovery. Palliative psychiatry has therefore been proposed as a complementary framework for selected patients with severe, refractory illness [90,91,92]. Rather than representing an alternative evidence-based treatment, it focuses on symptom relief, dignity, treatment burden, preservation of relationships, and goals defined with patients and their families. In CRS and UTRS, it may be considered in selected patients with substantial suffering or severe disability despite comprehensive evidence-based management. Treatment resistance alone, however, should not be considered an indication for palliative care.

Palliative psychiatry should be clearly distinguished from therapeutic nihilism. Its consideration requires careful attention to decision-making capacity, current preferences, autonomy, and previously expressed wishes, with involvement of family members or legally authorized representatives involved when capacity is impaired. Treatment goals should be reassessed over time, particularly in younger patients, to ensure that a palliative approach does not foreclose future recovery-oriented interventions [93]. However, the evidence base for palliative psychiatry in UTRS remains limited, being largely conceptual and grounded in clinical experience rather than controlled efficacy studies. Its use should therefore remain individualized and subject to regular review, with particular attention to treatment burden, patient preferences, and the preservation of future therapeutic options.

The absence of a standardized treatment algorithm does not preclude individualized management, but rather reflects the considerable heterogeneity of symptom profiles, comorbidities, previous treatment responses, treatment burden, and patient preferences. Individualized care should therefore be guided by these clinical and contextual factors rather than by treatment resistance alone.

Interpretation of our findings is limited by substantial heterogeneity in definitions of CRS and UTRS, minimum clozapine exposure, outcome measures, and augmentation strategies, which limits cross-study comparison and weakens evidence synthesis [35,40]. Although clozapine plus ECT has the strongest empirical support following clozapine failure, the available evidence remains insufficient to establish a standardized treatment pathway for CRS or UTRS. Future research should therefore standardize diagnostic reassessment and the assessment of adherence, clozapine dose, treatment duration, plasma exposure, and the adequacy of augmentation trials to improve comparability and distinguish true resistance from pseudoresistance. Multicenter cohorts and randomized trials should also use well-characterized CRS and UTRS populations, standardized symptom, cognitive and functional outcomes, and long-term follow-up including treatment burden and cumulative adverse effects [37,63]. Biomarkers, pharmacogenetic profiles, and neuroimaging measures require external validation and demonstrated clinical utility, including clarification of whether UTRS represents a biologically distinct subtype.

Several limitations should be considered. First, the patient was exposed to complex and rapidly evolving treatment regimens, including long-acting injectable paliperidone, multiple first- and second-generation antipsychotics, clozapine, pharmacological augmentation, anxiolytic treatment, and repeated courses of ECT. Some interventions were sequential, while others overlapped due to persistent psychosis, severe behavioral disturbances, catatonic symptoms, medical complications, and the need to ensure safety and nutritional support. This clinical complexity reflects the management of CRS and UTRS in clinical practice but limits causal attribution of clinical changes to a single intervention. In particular, it is not possible to determine with certainty which treatment contributed to transient improvements or subsequent deterioration, nor to separate the pharmacological effects from the influence of hospitalization, rehabilitation, changes in adherence, disease progression, and environmental confinement.

Second, this is a single clinical case, and the absence of standardized symptom assessments, systematic drug monitoring, and controlled treatment phases further restricts causal interpretation and generalizability. Therefore, the findings should not be interpreted as evidence of efficacy for any specific combination or sequence of interventions, and their generalizability is limited. However, the case highlights several issues that may be relevant to clinical practice. These include the need to reassess pseudoresistance, document treatment exposure and adherence, and distinguish different stages of treatment resistance. It also reflects the burden associated with increasing polypharmacy and coercive care, and the potential role of individualized multimodal and palliative approaches when sustained recovery cannot be achieved.

The search strategy represents an important limitation. Although the review focused specifically on CRS and UTRS, the use of these specific diagnostic terms may have resulted in the omission of relevant studies conducted under the broader concept of treatment-resistant schizophrenia or indexed primarily according to specific interventions. This may be particularly relevant for augmentation strategies targeting glutamatergic or other non-dopaminergic mechanisms. Therefore, the evidence summarized here should not be considered an exhaustive assessment of all therapeutic strategies potentially applicable to CRS or UTRS. Finally, a formal assessment of the risk of bias and methodological quality was not performed, as the review was designed to describe the breadth and characteristics of the available evidence, rather than to estimate the comparative effects of treatments. Since the evidence base was highly heterogeneous, the absence of a formal quality assessment limits the robustness of comparisons between interventions and should be considered when interpreting the findings.

## 5. Conclusions

This case illustrates the main clinical challenges of CRS and UTRS. Once clozapine pseudoresistance has been carefully excluded, treatment should go beyond switching antipsychotics and adopt an individualized, multimodal approach. Based on the available literature, ECT is the best-supported augmentation strategy after clozapine failure, while pharmacological augmentation and other neuromodulatory strategies remain limited by the heterogeneity of the evidence. Psychosocial rehabilitation should remain part of long-term management.

The review identified preliminary neuroimaging, glutamatergic, inflammatory, genetic, and epigenetic findings that may generate hypotheses about biological and clinical heterogeneity in CRS and UTRS. However, these findings are heterogeneous, insufficiently replicated, and no biomarker or neuroimaging measure has been validated as a diagnostic tool or for routine treatment selection. Future studies should standardize definitions, use adequately powered prospective trials, and assess functional and cognitive outcomes to determine whether these candidate findings can reliably define subgroups or predict treatment response. For patients who continue to experience severe disability despite comprehensive treatment, a palliative psychiatric approach focused on quality of life and treatment burden may be a useful complement to active care, although its role in CRS and UTRS remains insufficiently supported by empirical evidence.

## Figures and Tables

**Table 1:** Chronological summary of the clinical case.

Period	Location	Psychopathology	Complementary Tests	Treatment
April 2020–August 2022	Home	Persecutory delusions, self-neglect, irritability, suspiciousness, suicidal thoughts, social withdrawal		
September–October 2022	Acute Unit	Poorly systematized persecutory delusion, social withdrawal, affective indifference, apathy, alogia, abulia, lack of motivation, contained anger, referential thinking	Blood and urine tests without abnormalities; ECG without pathological findings; serology without abnormalities; EEG with normal cortical activity; brain CT without findings	Monthly paliperidone 150 mg + olanzapine 20 mg + quetiapine 200 mg. Referral to Outpatient Clinics and Day Hospital
October 2022	Home	Social withdrawal, lack of insight into illness		Monthly paliperidone 150 mg + olanzapine 10 mg
November–December 2022	Home	Paranoid ideation, hypervigilance, self-referential thinking, irritability, self-neglect		Monthly paliperidone 150 mg
January–April 2023	Home	Persecutory delusion, behavioral disturbances		Monthly paliperidone 300 mg
May 2023	Home	Unkempt appearance, blunted affect, abulia, self-referential thinking, paranoid ideation toward family	Blood and urine tests without abnormalities	Monthly paliperidone 300 mg + clozapine 200 mg
June 2023	Home	Persecutory delusion, irritability, self-neglect, insomnia		Monthly paliperidone 200 mg + clozapine 200 mg
July–September 2023	Home	Improvement in schedules and routines, appropriate behavior, resumed university studies	Hyperprolactinemia	Monthly paliperidone 150 mg + clozapine 200 mg + cariprazine 4.5 mg
October 2023–December 2024	Home	Self-care neglect, social withdrawal, irritability, self-referential thinking, paranoid ideation		
January–March 2025	Home	Confusion, unmotivated laughter, talking to self, hostility		
April 2025	Acute Unit	Behavioral disturbances, disorganized thinking, psychomotor blocking, word salad, auditory hallucinations, possession experiences	Blood and urine tests without abnormalities; brain MRI without abnormalities	Paliperidone 12 mg + olanzapine 30 mg + quetiapine 100 mg + zuclopenthixol 50 mg if crisis
April 2025	Acute Unit	Disability, dependency, support measures, and court order for admission to an appropriate facility were requested		
May–June 2025	Acute Unit	Behavioral disturbances, sexual disinhibition, apragmatism, abulia, blunted affect, cognitive decline		Paliperidone 12 mg + olanzapine 30 mg + diazepam 20 mg + 10 ECT sessions (L05)
July 2025	Long-stay Unit	Appropriate behavior, sparse speech, self-referent, and a tendency toward social withdrawal		Monthly paliperidone 200 mg + clozapine 250 mg + diazepam 30 mg
August–November 2025	Acute Unit	Confused, psychomotor blocking, echolalic speech, with threats and sexual propositions, even toward family members. Erratic and apragmatic behavior with disorganized thinking	Hyperprolactinemia; EEG with low-voltage cortical activity	Monthly paliperidone 200 mg + clozapine 300 mg + amisulpride 1200 mg + diazepam 15 mg + pimozide 4 mg
December 2025–February 2026	Internal medicine	Catatonia: immobile, rigid, trembling, no speech	PET-FDG with bilateral parietotemporal hypometabolism, predominantly left-sided, with bilateral frontal involvement; elevated prolactin (in the context of respiratory infection)	10 ECT sessions (DGX) + parenteral nutrition
March 2026	Long-stay unit	Social withdrawal, impoverished speech, cognitive decline, distant affect, irritability, brief agitation episodes with psychomotor agitation and apragmatic escapes, mystical-religious verbalizations, abnormal sensory filtering	Copper and ceruloplasmin normal. CSF without abnormalities, including antibodies, oligoclonal bands, and 14-3-3 protein. Onconeural antibodies negative. Metabolic disorder workup normal	Aripiprazole 15 mg + clozapine 75 mg + ECT (DGX) every 10 days
April 2026	Acute care	Psychomotor blocking, inability to abstract, lack of agency, frontal syndrome	Blood and urine tests without abnormalities	Risperidone 3 mg + clozapine 150 mg
May–September 2026	Long-stay Unit	Appropriate behavior, tendency toward social withdrawal and impoverished speech		Risperidone 2 mg + clozapine 200 mg + sodium valproate 800 mg

**Table 2:** Characteristics and main findings of the included studies.

Author (Year)	Country	Population	Study Design	Sample	Domain	Main Findings
Morrato et al. (2007) [13]	United States	Mixed	Retrospective pharmacoepidemiological study between 1998 and 2003	N = 55,481	Polypharmacy	Clozapine polypharmacy was common despite limited evidence supporting superior effectiveness and with increased risk of adverse effects
Barretto et al. (2009) [14]	Brazil	CRS	Preliminary controlled trial	N = 21	Psychosocial interventions (CBT)	CBT improved psychopathology, PANSS total score and quality of life, with benefits maintained at 6-month follow-up
Tiihonen et al. (2009) [15]	Finland	CRS	Systematic review and meta-analysis	5 studies (N = 161)	Pharmacological augmentation (lamotrigine)	Lamotrigine augmentation showed superiority over placebo for total, positive and negative symptoms
de Jesus et al. (2011) [16]	Brazil	CRS	Double-blind sham-controlled randomized trial	N = 17	Neuromodulation (rTMS)	Active rTMS improved general psychopathology but did not show clear benefit for auditory hallucinations, quality of life, or functioning
De Risio et al. (2011) [17]	Italy	CRS	Prospective open-label augmentation study	N = 16	Pharmacological augmentation (aripiprazole)	Reported clinical improvement with aripiprazole augmentation, providing an early signal in favor of this strategy
Repo-Tiihonen et al. (2012) [18]	Finland	CRS	Randomized crossover trial	N = 15	Pharmacological augmentation (olanzapine)	Clozapine monotherapy performed similarly to clozapine plus olanzapine, questioning routine polypharmacy
Shivakumar & Venkatasubramanian (2012) [19]	India	CRS	Case report with literature review	N = 1	Pharmacological augmentation (raloxifene)	Raloxifene was associated with clinical improvement in the reported case, but evidence remained very limited
Goldstein et al. (2015) [20]	New Zealand	Mixed	Cross-sectional proton magnetic resonance spectroscopy study	N = 58	Glutamatergic biomarkers	Differences in glutamatergic metabolites supported a possible glutamatergic basis for clozapine resistance
Seddigh et al. (2015) [21]	Iran	CRS	Case report	N = 1	Pharmacological augmentation (levothyroxine)	Levothyroxine appeared beneficial in a single case and was proposed as an experimental strategy with limited evidence
Srisurapanont et al. (2015) [22]	Thailand	CRS	Systematic review and meta-analysis	8 studies (N = 528)	Pharmacological augmentation (aripiprazole)	Aripiprazole improved psychopathology and metabolic outcomes with acceptable tolerability
Arumugham et al. (2016) [23]	India	CRS	Narrative review		Neuromodulation	ECT showed the strongest evidence among neuromodulatory interventions, whereas rTMS, tDCS and other techniques remained experimental
Kallel et al. (2016) [24]	Tunisia	CRS	Case series	N = 3	Neuromodulation (tACS)	Theta-rhythm transcranial alternating current stimulation showed preliminary improvement in negative symptoms, although evidence remained exploratory
Correll et al. (2017) [25]	United States	Mixed	Systematic overview of meta-analyses	29 studies (N = 19,833)	Pharmacological augmentation	Overall evidence quality was low, and no intervention reached a high-certainty recommendation
Kim et al. (2017) [26]	South Korea	CRS	Retrospective observational study	N = 7	ECT	ECT augmentation significantly improved symptoms, with response associated with treatment intensity and clozapine plasma levels
Kim et al. (2018) [27]	South Korea	Mixed	Clinical comparative study	N = 30	ECT	Clozapine plus ECT achieved greater symptomatic improvement than clozapine alone, particularly for persistent positive symptoms
McNabb et al. (2018) [28]	New Zealand	Mixed	Resting-state functional MRI study	N = 52	Neurobiology/Functional connectivity	Functional network disconnection was associated with treatment resistance, supporting its potential as a neuroimaging biomarker
Morrison et al. (2018) [29]	United Kingdom	CRS	Randomized assessor-blinded controlled trial	N = 487	Psychosocial interventions (CBT)	CBT showed mixed benefits, supporting a clinical signal but not robust universal efficacy
Siskind et al. (2018) [30]	Australia	CRS	Systematic review and meta-analysis	46 studies (N = 2163)	Pharmacological augmentation	Several strategies showed modest benefit, but the evidence was heterogeneous and limited
Wang et al. (2018) [31]	China	CRS	Systematic review and meta-analysis	18 studies (N = 1769)	ECT	ECT augmentation significantly improved clinical response compared with clozapine alone
Bartoli et al. (2019) [32]	Italy	CRS	Meta-analysis	12 studies (N = 693)	Pharmacological augmentation	Second-generation antipsychotic augmentation showed modest benefit for selected symptom domains but insufficient evidence for routine use
Iwata et al. (2019) [33]	Japan	UTRS	Cross-sectional 3T proton magnetic resonance spectroscopy study	N = 74	Neurobiology Glutamatergic biomarkers	Patients with UTRS showed altered glutamatergic metabolites in the putamen, supporting non-dopaminergic mechanisms of ultra-resistance
Lindenmayer et al. (2019) [34]	United States	UTRS	Pilot open-label clinical study	N = 21	Neuromodulation (tDCS)	Transcranial direct current stimulation (tDCS) produced modest improvements in auditory hallucinations and global psychopathology
Nucifora et al. (2019) [35]	United States	Mixed	Narrative review		Therapeutic strategies	Reviewed clinical characteristics, biological mechanisms and therapeutic options, highlighting the lack of high-quality evidence after clozapine failure
Petrides et al. (2019) [36]	United States	CRS	Prospective randomized study	N = 39	ECT	Adjunctive ECT plus clozapine produced significantly greater improvement than clozapine alone in a substantial proportion of patients
Wagner et al. (2019) [37]	Germany	CRS	Systematic review of systematic reviews (meta-review)	29 studies (N = 13,598)	Pharmacological augmentation	No pharmacological augmentation strategy demonstrated sufficiently robust evidence to become standard of care
Balcioglu et al. (2020) [38]	Turkey	CRS	Case report	N = 1	Pharmacological augmentation (long-acting aripiprazole)	Long-acting injectable aripiprazole improved adherence and clinical stability in a patient with CRS
Lin et al. (2020) [39]	Taiwan	Mixed	Narrative review		Pharmacological augmentation (sodium benzoate)	Sodium benzoate was proposed as a promising glutamatergic and antioxidant augmentation strategy, although clinical evidence remained limited
Campana et al. (2021) [40]	Germany	UTRS	Systematic review and meta-analysis	42 studies (N = 2731)	Definitions Methodology	Demonstrated marked heterogeneity in UTRS definitions and emphasized the need for standardized diagnostic criteria
Chakrabarti (2021) [41]	India	CRS	Narrative review		Pharmacological augmentation	Reviewed current management strategies and emphasized optimization of clozapine before considering augmentation or declaring treatment failure
Grover et al. (2021) [42]	India	CRS	Comparative observational study	N = 68	ECT	ECT augmentation improved symptoms in both CRS and non-CRS, supporting its role after clozapine failure
Ochi et al. (2022) [43]	Japan	Mixed	Structural neuroimaging study (MRI)	N = 100	Neurobiology Structural neuroimaging	Demonstrated structural brain abnormalities associated with clozapine resistance, supporting the hypothesis that CRS and UTRS represent a biologically distinct subtype of schizophrenia
Orsolini et al. (2022) [44]	Italy	CRS	Case report	N = 1	Pharmacological augmentation (brexpiprazole)	Brexpiprazole augmentation was associated with marked clinical improvement after inadequate response to clozapine
Poonia et al. (2022) [45]	India	CRS	Case series	N = 6	Pharmacological augmentation (amisulpride)	Amisulpride augmentation of clozapine was associated with clinically meaningful symptom improvement in patients with CRS and was generally well tolerated, although the evidence is limited by the uncontrolled case-series design
Zhu et al. (2022) [46]	China	CRS	Randomized, double-blind, placebo-controlled trial	N = 40	Pharmacological augmentation (amisulpride)	Amisulpride augmentation significantly improved overall psychopathology, positive symptoms, general psychopathology, language performance, and treatment response compared with placebo
Alp et al. (2023) [47]	Turkey	UTRS	Case report with literature review	N = 1	Neurobiology Genetics	Identified a KMT2D variant potentially associated with UTRS
Kitajima et al. (2023) [48]	Japan	Mixed	Cross-sectional structural MRI study	N = 85	Neurobiology Structural neuroimaging	UTRS was associated with reduced cortical gyrification and decreased medial parietal cortical surface area
Montgomery et al. (2023) [49]	United Kingdom	CRS	Narrative review		Pharmacological augmentation (cariprazine)	Cariprazine was proposed as a promising augmentation strategy, particularly for predominant negative symptoms
Pang et al. (2023) [50]	China	Mixed	Systematic review	32 studies (N = 1322)	Neurobiology Neuroimaging	Synthesized structural and functional neuroimaging abnormalities involving prefrontal, cingulate, frontostriatal and parietal networks
Peitl et al. (2023) [51]	Croatia	UTRS	Narrative review		ECT	ECT remains the biological intervention supported by the strongest evidence after clozapine failure
Tirupati (2023) [52]	Australia	UTRS	Narrative review Commentary		General management	Highlighted the absence of standardized treatment algorithms and emphasized individualized multimodal management
Yeh et al. (2023) [53]	Taiwan	CRS	Systematic review and network meta-analysis	35 studies (N = 1472)	Pharmacological augmentation & ECT	ECT ranked among the most effective augmentation strategies, whereas pharmacological interventions showed modest and heterogeneous benefits
Tuovinen & Hofer (2023) [54]	Finland	Mixed	Systematic review	18 studies (N = 648)	Neurobiology Functional neuroimaging	Resting-state fMRI consistently demonstrated abnormalities in functional connectivity involving the default mode, salience and frontoparietal networks
Mishra et al. (2024) [55]	India	CRS	Bayesian network meta-analysis	30 studies (N = 1336)	Pharmacological augmentation	Several augmentation strategies showed modest efficacy, although certainty of evidence remained low because of heterogeneity
Pathak et al. (2024) [56]	India	CRS	Case report	N = 1	Neuromodulation (accelerated tACS)	Accelerated transcranial alternating current stimulation was feasible and associated with preliminary clinical improvement
Bagali et al. (2025) [57]	India	UTRS	Case report	N = 1	Neuromodulation (continuous theta-burst stimulation)	Lateral occipital cortex stimulation produced improvement in refractory auditory and visual hallucinations
de Oliveira et al. (2025) [58]	Brazil	UTRS	Cross-sectional biomarker study	N = 49	Neurobiology Peripheral biomarkers	GFAP and neurofilament light chain differentiated UTRS from other schizophrenia subgroups and may represent promising biomarkers
de Pinho et al. (2025) [59]	Brazil	UTRS	Exploratory case–control study	N = 53	Neurobiology Immunology & Epigenetics	Immune dysregulation and LINE-1 abnormalities were associated with UTRS
Kawashima et al. (2025) [60]	Japan	Mixed	Retrospective observational cohort	N = 116	ECT	Long-term trajectories supported the effectiveness of clozapine and ECT, although substantial variability in response persisted
Mukherjee et al. (2025) [61]	India	CRS	Case series	N = 3	Neuromodulation (continuous theta-burst stimulation)	Continuous theta-burst stimulation showed encouraging preliminary results for persistent auditory hallucinations
Prodi et al. (2025) [62]	Italy	CRS	Prospective multicenter observational study	N = 45	Pharmacological augmentation (lurasidone)	Lurasidone augmentation improved predominantly negative symptoms and was generally well tolerated
Samara et al. (2025) [63]	Greece	Mixed	Systematic review and network meta-analysis	150 studies (N = 11,375)	Pharmacological augmentation	Pharmacological augmentation strategies demonstrated only modest overall benefits, with no universally superior intervention identified
Xiang et al. (2025) [64]	China	CRS	Randomized, parallel-group, controlled clinical trial	N = 16	Neuromodulation (Magnetic Seizure Therapy)	Magnetic seizure therapy (MST) was proposed as a promising alternative to ECT, with potential efficacy and a lower cognitive burden, although evidence remains limited
Wang et al. (2025) [65]	China	UTRS	Randomized controlled trial	N = 70	ECT	Modified ECT combined with clozapine showed greater clinical improvement than standard treatment in patients with UTRS
Armando et al. (2026) [66]	Indonesia	CRS	Systematic review	6 studies (N = 21)	Neuromodulation (Deep Brain Stimulation)	Deep brain stimulation (DBS) remains an experimental option for patients with extreme treatment resistance, with outcomes varying according to the stimulation target
Barros et al. (2026) [67]	Brazil	Mixed	Systematic review and meta-analysis	3 studies (N = 102)	ECT	Confirmed ECT as the augmentation strategy with the strongest evidence for patients with CRS
Dang et al. (2026) [68]	United States	CRS	Two case reports of xanomeline–trospium in CRS	N = 2	Pharmacological augmentation (xanomeline–trospium)	Adjunctive xanomeline–trospium produced limited clinical benefit, providing insufficient evidence to support routine use
Lin & Lane (2026) [69]	Taiwan	Mixed	Secondary analysis of a randomized controlled trial	N = 60	Pharmacological augmentation (sodium benzoate)	Sodium benzoate improved positive and negative symptoms, functioning and quality of life, supporting further evaluation as an adjunctive treatment

## Data Availability

No new data were created or analyzed in this study. Data sharing is not applicable to this article.

## Supplementary Materials

The following supporting information can be downloaded at: https://www.mdpi.com/article/10.3390/jcm15187264/s1, Table S1: Electronic search strategy used; Table S2: Distribution of included studies according to study design.
