# Supplementary material for: Beyond Clozapine Failure: Precision Approach and Palliative Psychiatry in Ultra-Treatment-Resistant Schizophrenia
Source: J Clin Med. 2026 Sep 18;15(18):7264. doi: 10.3390/jcm15187264 (PMC13607815; doi:10.3390/jcm15187264)
Supplement: Supplementary file 1 [file jcm-15-07264-s001.zip › jcm-4497289-supplementary.pdf]

Table S1. Electronic search strategy used.

Database	Date searched	Search strategy	Limits/filters
PubMed/MEDLINE	31 July 2026	(clozapine-resistant-schizophrenia OR ultra-treatment-resistant-schizophrenia) AND (psychopathology OR treatment OR neurobiology OR biomarker)	No filters applied
Embase	31 July 2026	(clozapine-resistant-schizophrenia/exp OR ultra-treatment-resistant-schizophrenia/exp) AND (psychopathology/exp OR treatment/exp OR neurobiology/exp OR biomarker/exp)	No filters applied
Web of Science	31 July 2026	(clozapine-resistant-schizophrenia OR ultra-treatment-resistant-schizophrenia) AND (psychopathology OR treatment OR neurobiology OR biomarker)	No filters applied

Note: The original database-specific search histories were not fully preserved. Therefore, it was not possible to reliably reconstruct platform-specific field labels, controlled vocabulary assignments, and complete line-by-line syntax. The table reports the search strategy based on the documentation available to the authors.

Table S2. Distribution of included studies according to study design.

Study design	N	Investigation included (references)
1. Meta-Analyses and Systematic Reviews	15 (26.3%)	[15,22,25,30,31,32,37,40,50,53,54,55,63,66,67]
2. Randomized controlled trial	8 (14.1)	[14,16,18,29,36,46,64,69]
3. Observational studies	16 (28%)	[13,17,20,26,27,28,33,34,42,43,48,58,59,60,62,65]
4. Case series	3 (5.2%)	[24,45,61]
5. Case report	8 (14.1%)	[19,21,38,44,47,56,57,68]
7. Narrative reviews	7 (12.3)	[23,35,39,41,49,51,52]
